# Optimized membrane filtration for high-purity red blood cell isolation from human and murine samples

**DOI:** 10.1080/07366205.2026.2699085

**Published:** 2026-07-20

**Authors:** Luis E. F. Almeida, Meghann L. Smith, Sayuri Kamimura, Kamille West-Mitchell, Zenaide M. N. Quezado

**Affiliations:** a Department of Perioperative Medicine, National Institutes of Health Clinical Center, National Institutes of Health, Bethesda, MD, USA; b Department of Transfusion Medicine, National Institutes of Health Clinical Center, Bethesda, MD, USA

**Keywords:** Leukoreduction, sickle cell, blood cells, filtration, membrane, red blood cell

## Abstract

Accurate experimental analysis of red blood cells (RBC) warrants its purification from other blood components as even low-level contamination by unwanted cell populations can introduce substantial artifacts. Conventional purification methods, such as density-based separation followed by removal of intermediate layers, often leave significant residual contamination. Other approaches, including affinity-based separation or flow-based sorting, achieve high purity but are limited by low throughput, high cost, and impracticality for processing large sample volumes. Filtration-based strategies using cellulose matrices have been widely employed; however, these approaches are time-consuming, sensitive to variability in matrix preparation across laboratories, and constrained by fixed pore structures inherent to the material. We developed an RBC purification method based on commercially available filter membranes. This approach proved simple, cost-effective, and required no specialized equipment. Importantly, membrane pore size can be readily selected to match the physical properties of the RBC targeted population, improving purification efficiency and reproducibility. Using this membrane-based filtration protocol, we achieved effective removal of contaminating cells in human and murine RBC samples. Overall, this method provides a robust and inexpensive RBC purification strategy that supports reliable downstream functional and biochemical analyses across a wide range of experimental applications especially for low volume samples.

## PLAIN LANGUAGE SUMMARY

Plain Language SummaryStudying red blood cells (RBCs) accurately requires isolating them from white blood cells (WBC) or platelets, because even small amounts of these other cells can interfere with laboratory experiments. Common purification methods, such as centrifugation, often leave behind unwanted cells. Other techniques, like magnetic sorting or cell sorting machines, can produce very pure RBCs but are slow, expensive, and not practical if you have many samples to process. Filtration through cellulose is another option, but it can be inconsistent, time consuming, and limited by the fixed pore sizes of cellulose fibers. We developed a simple, low cost method to purify small volumes of RBCs using readily available filter membranes. This approach does not require special equipment, and the membrane pore size can be chosen to match the size and flexibility of different types of RBCs. This improves the ability to remove WBCs and platelets effectively. Using this method, we successfully purified RBCs from both humans and mice. Overall, this technique provides a reliable, easy, and inexpensive way to obtain pure RBC samples for laboratory research.

## METHOD SUMMARY

Whole blood samples from human and murine sources were processed using a mixed cellulose ester membrane-based filtration protocol designed to purify red blood cells (RBCs) by removing white blood cells (WBCs) and platelets. Blood was passed through commercially available filter membranes with defined pore sizes selected to accommodate species-specific and disease-associated differences in RBC size and deformability. Filtration was performed under gravity or low-pressure conditions without the need for specialized equipment. Post-filtration samples were assessed for residual WBC and platelet content to determine leukoreduction and platelet removal efficiency. This approach was evaluated across samples with varying initial leukocyte counts and compared for robustness and applicability across species.

## 1. Introduction

In laboratory research and transfusion medicine, preparation of red blood cells (RBC) with minimal levels of white blood cells (WBC) contamination is critical. In clinical care, leukocyte filtration of large-volume red cell units (280–350 mL) is successfully achieved with leukoreduction filters using nonwoven synthetic fiber (polyester or polypropylene), which operate via depth filtration combined with adhesive and electrostatic interactions, enabling efficient reduction to <1 × 10^6^ leukocytes per unit. In turn, transfusion of leukoreduced RBCs reduces the incidence of transfusion-related reactions [1] and lowers the risk of transmission of leukocyte-associated infections [2]. In research settings, while researchers typically use smaller blood volumes, insufficient RBC purity (particularly due to residual WBCs) can compromise assay accuracy, and reduce reproducibility [3].

WBC contamination of RBC preparations, especially by neutrophils, can introduce substantial experimental artifacts that undermine data accuracy and interpretation [3]. One major artifact is misattribution of biological effects, whereby effects originating from contaminating WBC are incorrectly attributed to RBCs. Additionally, activated or damaged WBCs can release hydrolases, oxidases, or other enzymes that can damage RBC proteins, and therefore their function, during the purification process. Consequently, minimizing non-RBC cellular contamination is essential when preparing blood samples for experimental use. A commonly used RBC purification technique involves centrifugation, buffy coat removal, and repeated washes of packed RBC. Albeit simple, this approach is ineffective because neutrophils co-precipitate with RBCs during centrifugation [3], resulting in significant WBC contamination – typically 150–400 WBCs per 10^6^ RBCs [4]. Cellulose-based filtration is a simple and widely used method for purifying RBCs, particularly from small-volume samples, and has long been recommended as a standard practice in RBC research [5,6]. However, it is time-consuming (filtration time between 10 and 17 min per sample), subject to variability in cellulose bed preparation, and constrained by fixed pore sizes, limiting its applicability across species and in samples containing RBCs with altered deformability, such as spherocytosis, elliptocytosis, ovalocytosis, or sickle cell disease (SCD), where filtration efficiency and RBC recovery are often reduced [5,6]. Other purification approaches including density gradient centrifugation, magnetic-activated or fluorescence-activated cell sorting can produce highly pure RBC populations, however, these methods require specialized equipment and are often limited by low throughput and high cost, particularly when processing large sample volumes [7].

We describe a practical, low-cost RBC purification method using commercially available filter membranes, which was optimized for small-volume RBC preparations, particularly in experimental settings where standard blood bank leukoreduction systems are not applicable (e.g. murine samples or limited-volume studies). We evaluated the method’s performance using human and murine samples with varying WBC counts.

## 2. Material and methods

### 2.1. Regulatory approvals and ethical disclosure

The National Institutes of Health (NIH) Clinical Center Animal Care and Use Committee approved all animal protocols. The NIH Institutional Review Board approved the human research protocol (number 99-CC-0168: NCT00001846). Human blood samples were obtained from healthy blood donors after written informed consent.

### 2.2. Red blood cell purification from animal blood samples

We used a humanized mouse model of SCD (HbSS) that expresses human sickle hemoglobin and recapitulates phenotypes of human SCD including reduced RBC deformability and leukocytosis [8,9]. Control mice (HbAA) express human hemoglobin A. Age (>20 weeks) and sex-matched male and female mice were included in all experimental groups. We collected approximately 1 mL of blood from mice anesthetized with isoflurane via cardiac puncture.

**Figure 1.**
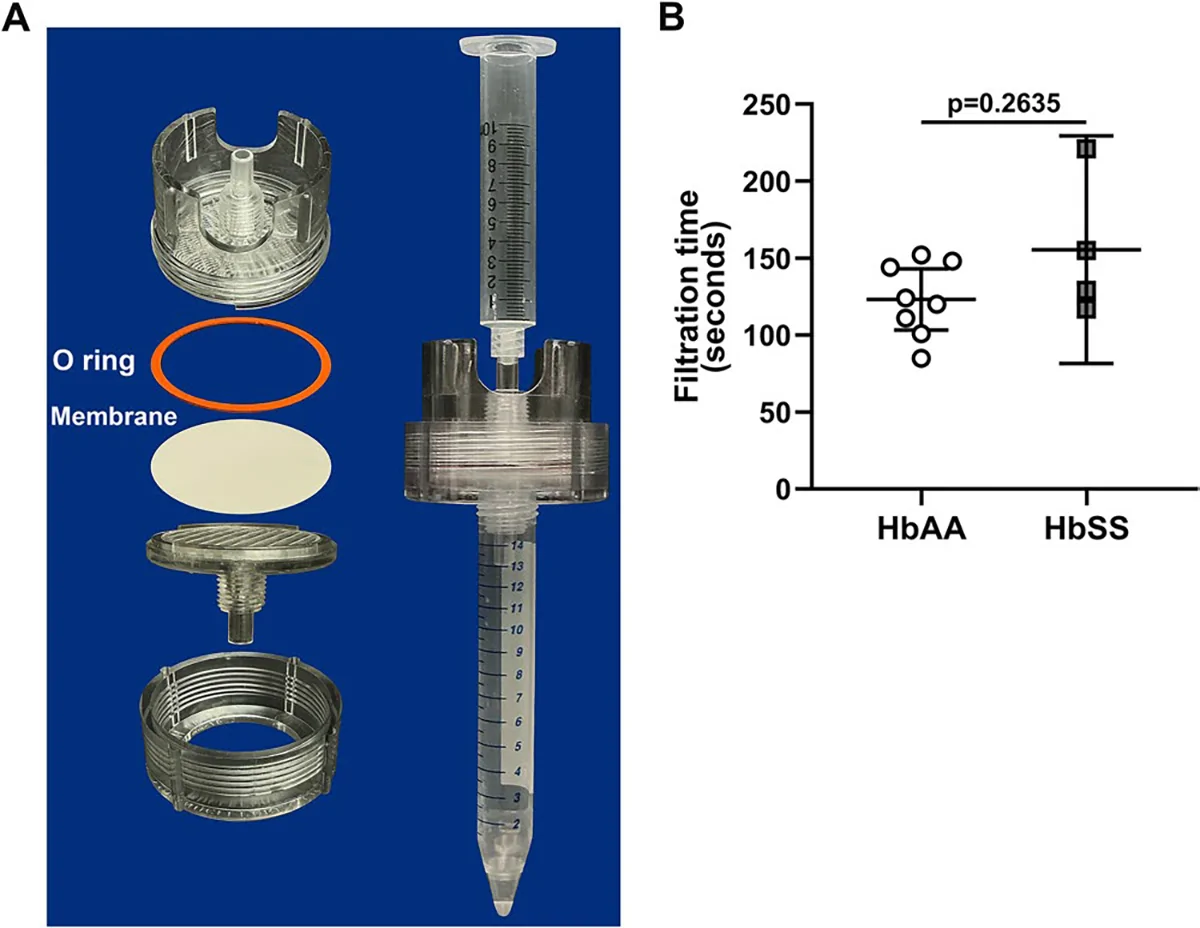
Experimental set up for whole blood filtration through a mixed cellulose ester (MCE) membrane. (A) The blood sample, diluted in phosphate-buffered saline at 4 °C, is poured into an inverted syringe positioned above the membrane holder. The blood sample flows through the holder containing a 47-mm mixed cellulose ester (MCE) membrane spontaneously, with pore size selected according to red blood cell size and deformability. When necessary, the syringe plunger is gently inserted to apply minimal pressure and facilitate passage of the sample through the membrane or to push any blood remaining in the filter dead space to increase recovery. Purified red blood cells are collected in a vial below the holder, while white blood cells and platelets are retained on the membrane. (B) Filtration times in seconds measured from when blood was poured into the membrane to collection of filtrate. Data show individual sample measurements with overlaid bars representing the least squares means and 95% confidence intervals.

Mouse samples were transferred to 10 mL tubes, diluted to 2 mL with phosphate-buffered saline (PBS) at 4 °C, and a complete blood count (CBC) was obtained with Heska Element HT5 analyzer (Antech Diagnostics). Samples were further diluted to 8 ml with PBS at 4 °C and placed on ice. Mixed cellulose ester (MCE) membranes (Tisch Scientific, #SF14634 and #SF14633) were mounted in a 47 mm Whatman membrane holder (Fisher Scientific, #420400), Figure 1. Visual inspection of the membrane to ensure correct fitting and sealing with the O-rings from the holder was essential. The membrane was pre-wetted by keeping the holder in a vertical position (with the intake side pointed downward) and perfusing 10 mL of PBS at 4 °C with a 10 mL syringe (the holder was perfused from bottom to top to facilitate air bubbles removal). The holder was then placed in a horizontal position, the syringe was disconnected, the piston removed, and the syringe body (without its piston) reconnected to the holder. Excessive PBS was drained by inverting the system to the vertical position (exit outlet downward) and placed into a 15 mL tube. Diluted blood was poured into the inverted syringe and filtration started spontaneously. The syringe piston was introduced carefully and used to gently push the sample through the membrane to finish filtration. Filtration time was measured using a stopwatch from the time blood was poured into membrane to collection of filtrate. We used 3 μm MCE membrane pore sizes for HbAA and 5 μm for HbSS mice. Filtered blood was precipitated with centrifugation (1,000 g, 5 min), supernatant was aspirated, and volume adjusted to 2 mL with PBS and another CBC was obtained to calculate purification efficiency. Following purification, RBCs can be washed and resuspended in the buffer of choice for subsequent experiment.

RBC recovery was determined by measuring an RBC count before and after filtration. Briefly, after RBC count, 1 mL of whole blood was diluted with PBS at 4 °C and filtered as described. Following filtration, RBCs were pelleted (500 × g, 5 min), resuspended in 2 mL of PBS, and a new RBC count was obtained. Total RBC numbers before and after filtration were calculated from the measured RBC concentration (×10^6^/μL) and sample volumes (1 mL pre-filtration; 2 mL post-filtration). Recovery was expressed as the percentage of total RBCs recovered after filtration relative to the initial RBC count.

RBC morphology was examined by evaluation of blood smears prepared and visualized as described [10]. Briefly, samples were diluted with PBS to Hb = 2.4 (± 0.1) g/dL and 3 μL were smeared on a glass slide. After air drying, slides were submerged sequentially in methanol (30 sec), Wright-Giemsa stain (3 min) and Giemsa-Buffer (6 min). The latter was prepared by mixing Wright-Giemsa stain (Millipore-Sigma #64571–75) with phosphate buffer pH 6.4 (Millipore-Sigma, #1217–75) (1:5 ratio, respectively). Slides were shaken in double-distilled water and left submerged for 6 min to remove excessive background. After air-drying, slides were visualized with an oil-immersed 100x objective in an upright microscope. Two slides were prepared from each sample (before and after filtration) and five pictures were taken from each slide with CaptaVision+ (Accu-Scope, Inc).

RBC membrane fragility was assessed by measuring sensitivity to hemolysis induced by sodium chloride 0.4%. After blood collection an aliquot (400 μL) was separated, mixed with 1 ml of PBS and placed in ice. The leftover blood volume was diluted with cold PBS and filtered as indicated. Afterwards, both fractions (pre- and post-filtration) were precipitated by centrifugation (1000 g, 5 min), washed with Hank’s balanced salt solution with Ca^+2^/Mg^+2^ and diluted to hemoglobin 2.4 ± 0.1 g/dL. A volume (18 μL) from each pre- and post-filtration fractions was added to 1 ml of sodium chloride 0%, 0.4% or 0.9% (g/v). Sodium chloride solutions were prepared in double-distilled water. Samples were allowed to lysis for 30 min at 37 °C. Remaining cells were precipitated (1000 g, 10 min) and 200 μL of supernatant was pipetted in duplicate in a clear 96-well plate. Free hemoglobin was assayed by absorbance at 540 nm. Results were analyzed by subtracting absorbance observed at 0.9% NaCl (0% lysis, blank) and normalizing to results obtained at 0% NaCl (100% lysis).

### 2.3. Red blood cell purification from human blood samples

Human blood was collected in Anticoagulant Citrate Dextrose solution (3 males, 64–74 years old, Caucasians) or in EDTA Vacutainer^®^ tubes (3 males, 2 females, 29–65 years old, 4 Caucasians, 1 Asian). Samples were centrifuged (430 g for 20 minutes) and platelet-rich plasma was discarded. Blood volume was reconstituted to its original volume with PBS. An aliquot (1.5 mL) was further mixed with 0.5 ml PBS and a CBC was obtained. Blood samples (2 mL) were further diluted to 8 mL with PBS at 4 °C and kept on ice. Diluted blood was filtered through 5 μm MCE membranes as described above. Filtered RBC were precipitated (1000 g, 10 min), resuspended into 2 mL PBS and another CBC was obtained. Removal of most platelets as a platelet-rich plasma was helpful to reduce platelet contamination. Samples from patients with conditions associated with changes in RBC size or properties, such as SCD or spherocytosis, may require membranes of different pore sizes, which need to be determined according to intended application.

### 2.4. RBC purification efficiency

Leukoreduction efficiency was calculated as [(WBC before −WBC after)/WBC before] × 100. Platelet reduction efficiency was similarly calculated, as the percentage of residual platelets remaining after filtration relative to pre-filtration values.

### 2.5. Assay of pro-MMP9

We evaluated leukoreduction by assaying mouse pro-MMP9 with a commercial ELISA kit (Abcam, #ab100732) before and after filtration. Unless otherwise indicated, experiments were carried out at room temperature. Samples were diluted so that their results fell within the standard curve provided by the manufacturer. After blood collection, volume was adjusted to 2 mL with PBS and a CBC obtained to correlate pro-MMP9 levels with RBC and WBC counts. An aliquot was prepared by diluting 3 μL of whole blood in 1,497μL of assay diluent (500-fold dilution). Samples were filtered as described and collected cells were diluted with PBS to 2 mL. Another CBC was obtained to document WBC removal. To increase our pro-MMP9 detection sensitivity, a much higher volume of filtrated blood was processed by mixing a 30 μL aliquot with 270 μL of diluent (10-fold dilution). Samples and standards (100 μL each) were loaded in duplicates and all subsequent procedures followed instructions. These procedures allowed us to assay samples containing approximately 50-fold higher sample volume after filtration than before.

### 2.6. Intracellular calcium detection

Intracellular calcium was imaged with Fluo-4 as described [10,11]. Briefly, purified RBCs were washed twice with Hank’s balanced salt solution (HBSS, without Ca^+2^/Mg^+2^), diluted to 2 mL and a CBC was obtained (Element HT5, Antech Diagnostics). A volume containing 1.5×10^9^ RBCs was pipetted into a new vial and its volume completed to 1.5 mL with HBSS. Samples were loaded with 10 μM fluo-4 AM (1h, 37 °C, in the dark) (final DMSO = 1.0%) (Thermo Fisher, #F14201). This protocol exposes equal number of RBCs to fluo-4 during loading phase so that differences in fluorescence intensity could be attributed to genotype or treatment, rather than to unequal fluorophore loading. Cells were precipitated, washed twice with HBSS (with Ca^+2^/Mg^+2^) and diluted to 1% (±0.1%) hematocrit using a CBC to record RBC numbers. Samples were distributed in duplicates (200 μL/well) into a black 96 well plate and fluorescence (Ex: 495; Em: 520) was acquired every minute for 2 hrs in a 37 °C plate reader. Although samples from each genotype had equal hematocrit (1 ± 0.1%) because HbAA and HbSS RBCs have different mean corpuscular volumes each genotype contained different numbers of RBCs [10,11]. Therefore, the recorded fluorescence was adjusted to 10^6^ RBCs calculated from the RBC counts.

Intracellular calcium was quantified by measuring fluorescence signal at t = 1min (baseline calcium), the rate of calcium accumulation between 20 (F_20_) and 40 min (F_40_) [Carate = (F_40_ – F_20_)/20] and the final (endpoint) calcium levels (t = 100min).

### 2.7. Red blood cell population analysis using flow cytometry

CBCs were obtained before and after sample filtration. A volume containing 10 × 10^6^ RBCs was pipetted into a new vial and diluted with 500 μL of Hank’s balanced salt solution (HBSS with Ca^+2^/Mg^+2^). Samples were labeled with Calcein-FITC (Invitrogen #C3100MP), TER119-APC (BioLegend #116212), CD71-AlexaFluor700 (Invitrogen #56–0711-82), CD41a-APCeFluor780 (Invitrogen #47–0411-82) and CD45-PE-Cyanine5.5 (Invitrogen #35–0451-82) and incubated for 30 min (protected from light). Excess markers were removed by centrifugation (1,000 g for 5 min) and cells resuspended into 500 μL HBSS (with Ca^+2^/Mg^+2^) for acquisition. All markers were optimized and used per manufacturers’ recommendations. Two million events were collected per sample using a CytoFlex S (Beckman Coulter). Single staining vials, Fluorescence Minus One (FMOs) and unstained samples were included as controls. Flow cytometry results were analyzed using FlowJo^™^ Software (v10.10, BD Life Sciences). Gating strategies excluding dead cells and doublets, were consistently applied to all runs, and data were generated by comparing before and after filtration staining taking into account false positives from individual FMOs.

### 2.8. Statistical analysis

Human samples were analyzed using a two-tailed paired Student’s t-test. Mouse data were analyzed by fitting a mixed-effect model using Restricted Maximum Likelihood (REML) with genotype (HbAA, HbSS) and time of filtration (pre or post filtration) included as fixed effects and individual mouse as a random factor. Continuous variables are presented as least square means and 95% confidence interval (unless otherwise indicated). Measurements falling at or below the blood analyzer reported detection thresholds were assigned the lowest quantifiable value and used for the analysis. Mouse calcium assay data were analyzed using the Welch’s t test. Model fit diagnostics were examined to confirm that assumptions were adequately met. Post hoc pairwise comparisons were adjusted using the Bonferroni correction, and adjusted p values are reported. A p value < 0.05 was considered statistically significant. Statistical analyses were performed in GraphPad Prism 10.2.1 (GraphPad Software, Boston, MA) or SigmaPlot (v. 15, Palo Alto, CA).

## 3. Results & discussion

### 3.1. Method optimization

After preliminary experiments evaluating membranes of various materials, including nylon, cellulose acetate, polytetrafluoroethylene, polyethersulfone, polypropylene, and Nuclepore track-etched membranes, with multiple diameters (13, 25, and 47 mm) and pore sizes, we chose MCE membranes as these yielded the most time-efficient RBC purification with both sides being suitable for filtration (Figure 1A). Optimal filtration efficiency and purity were achieved using a 5 μm pore size for human blood samples and 3–5 μm pore sizes for mouse blood samples. In addition, sample dilution with PBS at 4 °C consistently yielded the best results. Overall, the filtration time was approximately 134 seconds (95% confidence interval, CI: 111 to 157 seconds), Figure 1B.

Leukocyte reduction in transfusion medicine is typically achieved using nonwoven synthetic fiber filters composed of materials such as polyester or polypropylene. These systems use depth filtration and physicochemical interactions to selectively retain leukocytes while allowing erythrocytes to pass, achieving residual leukocyte counts below 1×10^6^ per unit. In contrast, the MCE membranes used in this study operate via size-based exclusion as thin microporous membranes. While suitable for small-volume, cost-effective applications, these membranes differ from clinical leukoreduction filters in structure, mechanism, and scalability. Our membrane-based filtration technique offers practical advantages for smallvolume research laboratory applications over cellulose-based systems, density gradient centrifugation, and magnetic-activated or fluorescence-activated cell. One potential significant advantage is increased operational efficiency as sample filtration times are reduced from approximately 10 to 17 minutes using cellulose-based systems [5] to an average of 134 seconds using MCE membrane (Figure 1B). Additionally, this method obviates the need for high-capacity centrifugation and costly supplies. A critical aspect of the protocol involves precise placement of the MCE membrane within the filtration holder to prevent leakage and ensure consistent flow. Compared to cellulose filters, membrane filtration achieves higher flow rates, enabling faster sample processing. Importantly, the availability of membranes with variable pore sizes allows for customization across different RBC sizes, species, and sample types. However, because filtration is also influenced by RBC deformability and structural heterogeneity, this approach may not be suitable for all RBC morphologies or pathological conditions. Nevertheless, this method represents a practical advantage over cellulose-based filtration, where pore size is inherently fixed by the fiber matrix, but does not fully overcome limitations related to altered cell deformability.

### 3.2. Human red blood cell filtration

**Figure 2.**
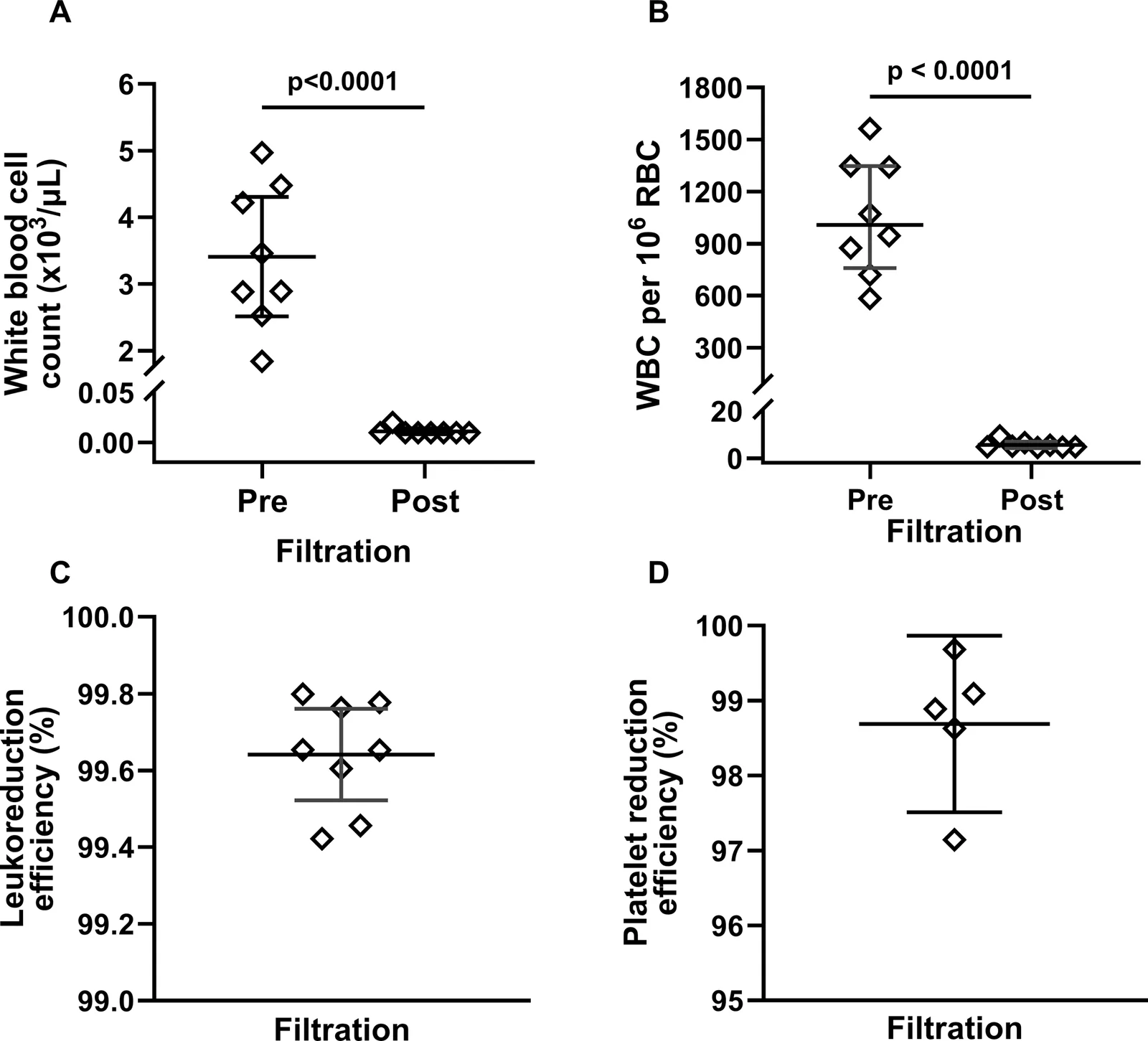
Red blood cell purification of human blood samples. Data are shown as scatter dot plots, illustrating individual human blood donor measurements, with overlaid bars representing the least squares means and 95% confidence intervals. (A) Following the removal of platelet-rich plasma and blood dilution, the mean white blood cell count (WBC) was 3.4×10^3^/μL [2.5, 4.3 ×10^3^/μL (95% CI)] and after filtration it was conservatively averaged as 0.01×10^3^/μL [0.0083, 0.0142 ×10^3^/μL (95% CI)] as 3 out of 8 samples reached the theoretical detection limit of the analyzer (0.00 ×10^3^ WBC/μL). B. Membrane filtration markedly reduced the WBC to red blood cell (RBC) ratio from 1,056 WBC per 10^6^ RBCs [773, 1,339 (95% CI)] to 6 WBC per 10^6^ RBCs [4, 7 (95% CI)], p < 0.0001. (C) Membrane filtration showed a leukoreduction efficiency of 99.6% [95% CI: 99.5–99.8%] and (D) a platelet reduction efficiency of 98.7%. N = 8. Platelet reduction was calculated only in 5 of the 8 samples.

We first examined the efficacy of membrane-filtration in human blood samples. We measured WBC, RBC, and platelet before (after platelet-rich plasma removal) and after membrane filtration (Figure 1) using an automated analyzer to evaluate the effect of filtration on sample purity. Following platelet-rich plasma removal and blood dilution (material and methods), the mean WBC was 3.4×10^3^/μL [2.5–4.3×10^3^/μL (95% CI)], Figure 2A. After membrane filtration, 3 out of 8 samples yielded zero WBC. For samples, with WBC counts at the analyzer’s detection threshold (reported as 0.00 ×10^3^ WBC/μL), we conservatively assigned the lowest quantifiable value reported by the analyzer (0.01 ×10^3^ WBC/μL) for statistical analysis. Using this approach, post-filtration human samples were estimated to contain 0.01 × 10^3^ WBC/μL (95% CI: 0.0083–0.0142 × 10^3^ WBC/μL). Because absolute WBC counts may be affected by sample dilution during processing, the WBC:RBC ratio provides a more robust measure of sample purity. Membrane filtration markedly reduced the WBC to RBC ratio from 1,056 (773–1,339, 95% CI) WBC per 10^6^ RBCs to 6 (4–7) WBC per 10^6^ RBCs, p < 0.0001, Figure 2B. This corresponds to a leukoreduction efficiency of 99.6% (99.5–99.8%), Figure 2C. Platelet counts before and after filtration showed a platelet reduction efficiency of 98.7% (97.5–99.9%), Figure 2D.

### 3.3. Murine red blood cell filtration

**Figure 3.**
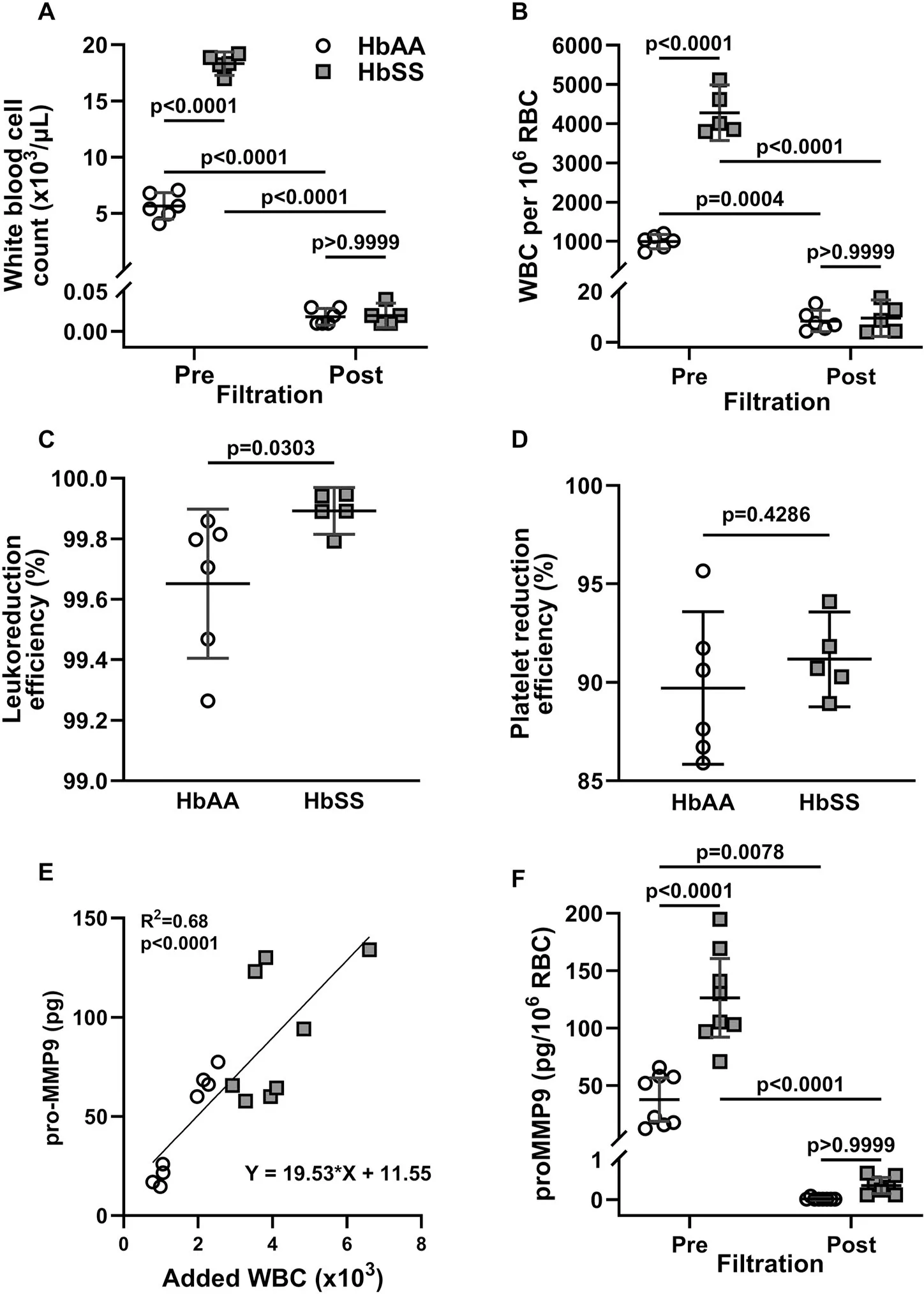
Red blood cell purification of murine blood samples. Data are shown as scatter dot plots, illustrating individual mouse measurements, with overlaid bars representing the least squares means and 95% confidence intervals. (A) Before filtration (after plasma and buffy-coat removal and dilution as described), samples from HbSS mice (N = 5) displayed significant leukocytosis compared with HbAA mice (N = 6). After membrane filtration, the white blood cell (WBC) count was 0.02 ×10^3^/μl (0.01–0.03, 95% CI) in HbAA and 0.02 ×10^3^/μl (0.01–0.04, 95% CI) in HbSS and was not statistically significant between genotypes (p > 0.9999). (B) Membrane-filtration markedly reduced the WBC to RBC from 994 (811–1178) WBCs per 10^6^ to 8 (4–13) WBC per 10^6^ RBCs in HbAA mice (N = 6) and from 4279 (3570–4988) WBCs per 10^6^ RBCs to 10 (2–17) WBCs per 10^6^ RBCs, in HbSS animals (N = 5). (C) The reductions in WBC to RBC ratios reflect a leukoreduction efficiency of 99.7% (99.4–99.9%) in HbAA and of 99.9% (99.8–100%) in HbSS. (D) The platelet reduction efficiency was 89.7% (85.8–93.6%) in HbAA and of 91.2% (88.8–93.6%) in HbSS. (E) Linear regression can estimate WBC counts and the purity (RBC/WBC ratio) of filtered and unfiltered samples, (N = 8 for HbAA and N = 8 for HbSS). (F) After filtration, proMMP9 levels were below the assay’s detection limit, indicating that HbAA (N = 8) and HbSS (N = 8) filtered samples had less than 16 (14–18) WBCs per 10^6^ RBC.

To evaluate the applicability of the filtration method across species and to examine its performance in samples with normal (HbAA) and elevated (HbSS) WBC counts (Figure 3), we conducted subsequent experiments using mouse blood. Before filtration (after plasma and buffy-coat removal), samples from HbSS mice still displayed higher WBCs compared with HbAA mice (p < 0.0001, Figure 3A). After membrane filtration all samples had mean WBC count 0.02 (0.01–0.03, 95% CI) ×10^3^/μL. In HbAA samples, membrane-filtration reduced WBC to RBC ratio from 994 (811–1178, 95% CI) WBC per 10^6^ RBCs to 8 (4–13) WBC per 10^6^ RBCs, p =0.0004, Figure 3B. In HbSS, membrane filtration reduced WBC to RBC ratio from 4279 (3570–4988) WBC per 10^6^ RBCs to 10 (2–17) WBC per 10^6^ RBCs, p < 0.0001, Figure 3B. These reductions corresponded to leukoreduction efficiencies of 99.7% (99.4–99.9%) in HbAA and 99.9% (99.8–100%) in HbSS mice, Figure 3C. Additionally, platelet reduction efficiency was 89.7% (85.8–93.6%) in HbAA and 91.2% (88.8–93.6%) in HbSS mice, Figure 3D.

#### 3.3.1. Levels of proMMP-9 become undetectable after murine blood filtration

To explore other approaches for estimating WBC contamination in filtered samples, we measured levels of proMMP9, the inactive precursor of matrix metalloproteinase-9 (MMP9). ProMMP-9 is predominantly stored in WBCs, particularly neutrophils, and its concentration correlates with WBC counts [12], Figure 3E and 3F. While HbSS samples exhibited elevated proMMP9 levels (Figure 3E), after normalization to respective WBC counts (expressed per 10^3^ WBC), HbAA and HbSS mice had comparable proMMP9 content per WBC: 25.45 (20.42–30.49) and 22.45 (15.95–28.95) pg proMMP9/10^3^ WBC, mean (95% CI), for HbAA and HbSS respectively, p = 0.4019. This indicates that higher proMMP9 in HbSS samples reflected leukocytosis rather than increased proMMP9 per leukocyte. Given the strong correlation between proMMP-9 levels and WBC counts (r = 0.82, p < 0.0001, Figure 3E), we employed linear regression to estimate WBC counts and the purity (RBC/WBC ratio) of filtered samples. Despite adding a 50-fold increase in RBC count to increase detection (see material and methods), after filtration, 7 out 8 HbAA samples had undetectable proMMP9 and we conservatively assigned the assay’s detection limit value to those samples (0.01 pg proMMP9/10^6^ RBC). In HbSS samples after filtration, values were 0.35 (0.15–0.56, 95% CI) pg proMMP9/10^6^ RBC. Using the average content of proMMP9/10^3^ WBC (Figure 3E), we calculated that HbAA and HbSS filtered samples had less than 16 (14–18, 95% CI) WBCs per 10^6^ RBC, Figure 3F.

Together these data indicate that standard removal of plasma and buffy coat leaves substantial WBC contamination in the packed RBC fraction, posing critical limitations for protein- and gene-amplification techniques, particularly RBC-focused “omics” analyses. Our membrane filtration method achieved robust leukodepletion efficiency (>99%) accompanied by substantial platelet reduction (approximately 90%), yielding highly purified RBC preparations. Although the mechanism of WBC membrane retention was not examined, it is likely analogous to the cellulose-based filtration mechanism described by Beutler and Gelbart, in which larger and stiffer WBCs are physically retained by the membrane, while more deformable RBCs traverse the pores [6]. Notably, despite being smaller than both WBC and RBC, platelets were markedly reduced by membrane filtration, raising the possibility that platelet activation and subsequent adhesion to the membrane or to retained WBCs contributed to their depletion.

### 3.4. Effects of membrane filtration on the morphology and composition of red blood cell population

**Figure 4.**
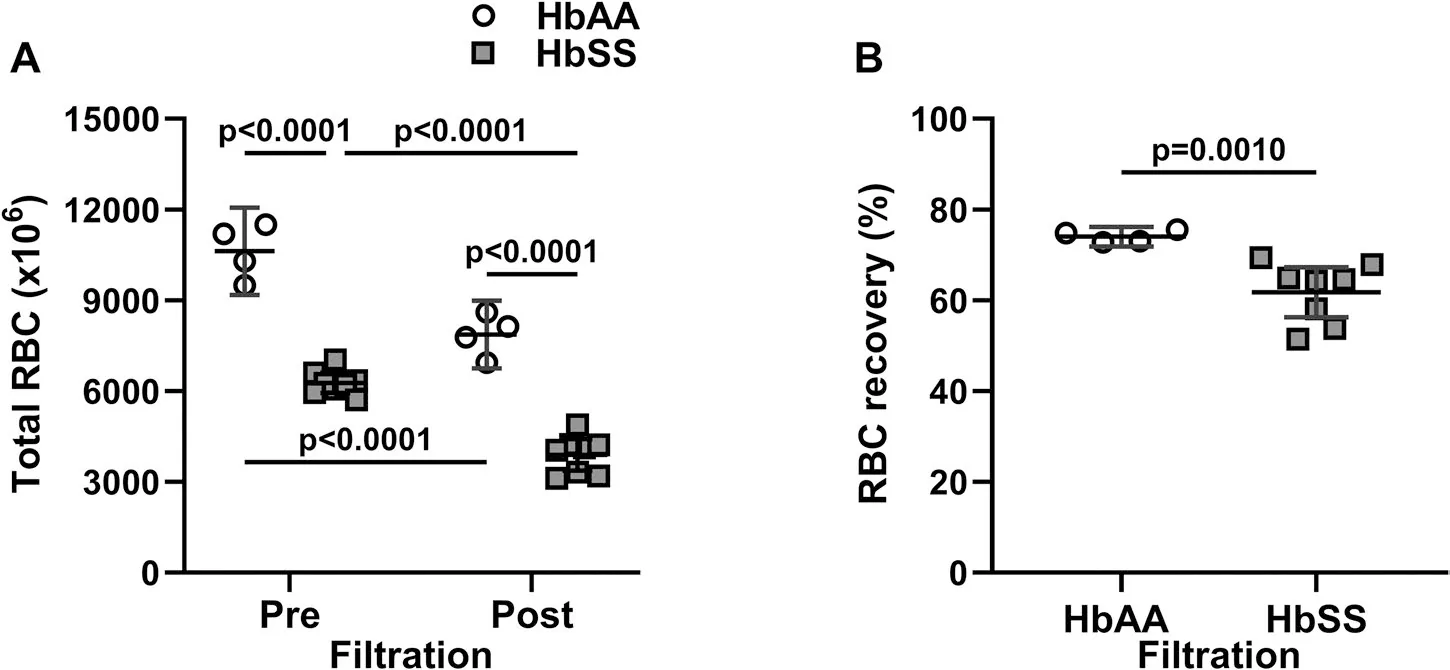
Red blood cell recovery. Data are shown as scatter dot plots, illustrating individual mouse measurements, with overlaid bars representing the least squares means and 95% confidence intervals. (A) Total red blood cell (RBC) count before (pre) and after (post) filtration in HbAA (N = 4) and HbSS (N = 8) mice. (B) With MCE filtration, RBC recovery was lower in HbSS samples compared with HbAA (p = 0.0010) as approximately 74% of RBCs from HbAA samples and 62% of HbSS RBCs were recovered.

**Figure 5.**
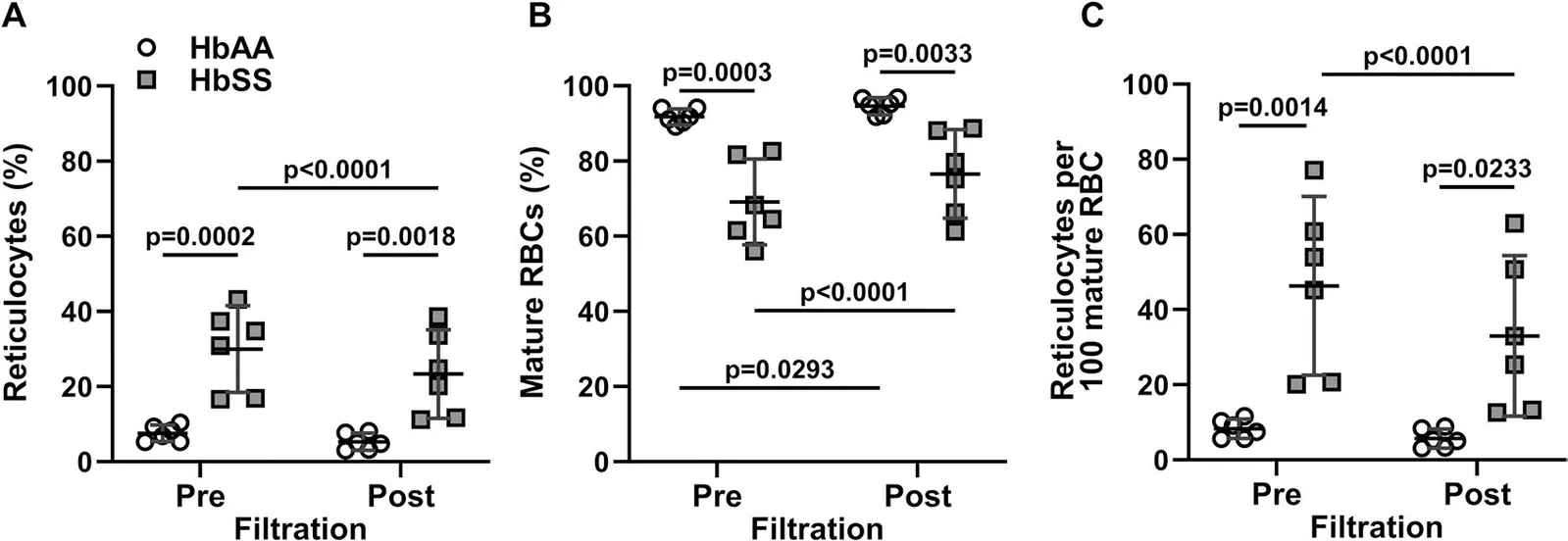
Reticulocyte/Mature RBC composition before and after filtration. Data are shown as scatter dot plots, illustrating individual mouse measurements, with overlaid bars representing the least squares means and 95% confidence intervals. (A) Using flow cytometry, the total red blood cells (RBC) population was defined as Ter119^+^ cells, while immature RBCs were defined as Ter119^+^ and CD71^High^and mature RBCs as Ter119^+^ and CD71^Low^ (see material and methods). (A) Before filtration, in HbAA blood, immature RBCs accounted for 7.6% (5.3, 9.7%) [mean (95% CI)] and in HbSS for 30% (18.4, 41.5%) of circulating RBCs, Figure 4A. In HbAA, there was a trend for reduction in immature RBCs that did not reach significance. Conversely, in HbSS blood there was a reduction in immature RBCs to 23% (12, 35.2%). (B) There were corresponding modest but significant increases in the percentage of mature RBCs both in HbAA and HbSS mice, indicating an enrichment of the mature RBCs population. (C) Membrane filtration significantly reduced the ratio of reticulocyte to mature RBC in HbSS (p < 0.0001) but not in HbAA mice (p = 0.6655). HbAA, N = 6 and HbSS, N = 6.

**Figure 6.**
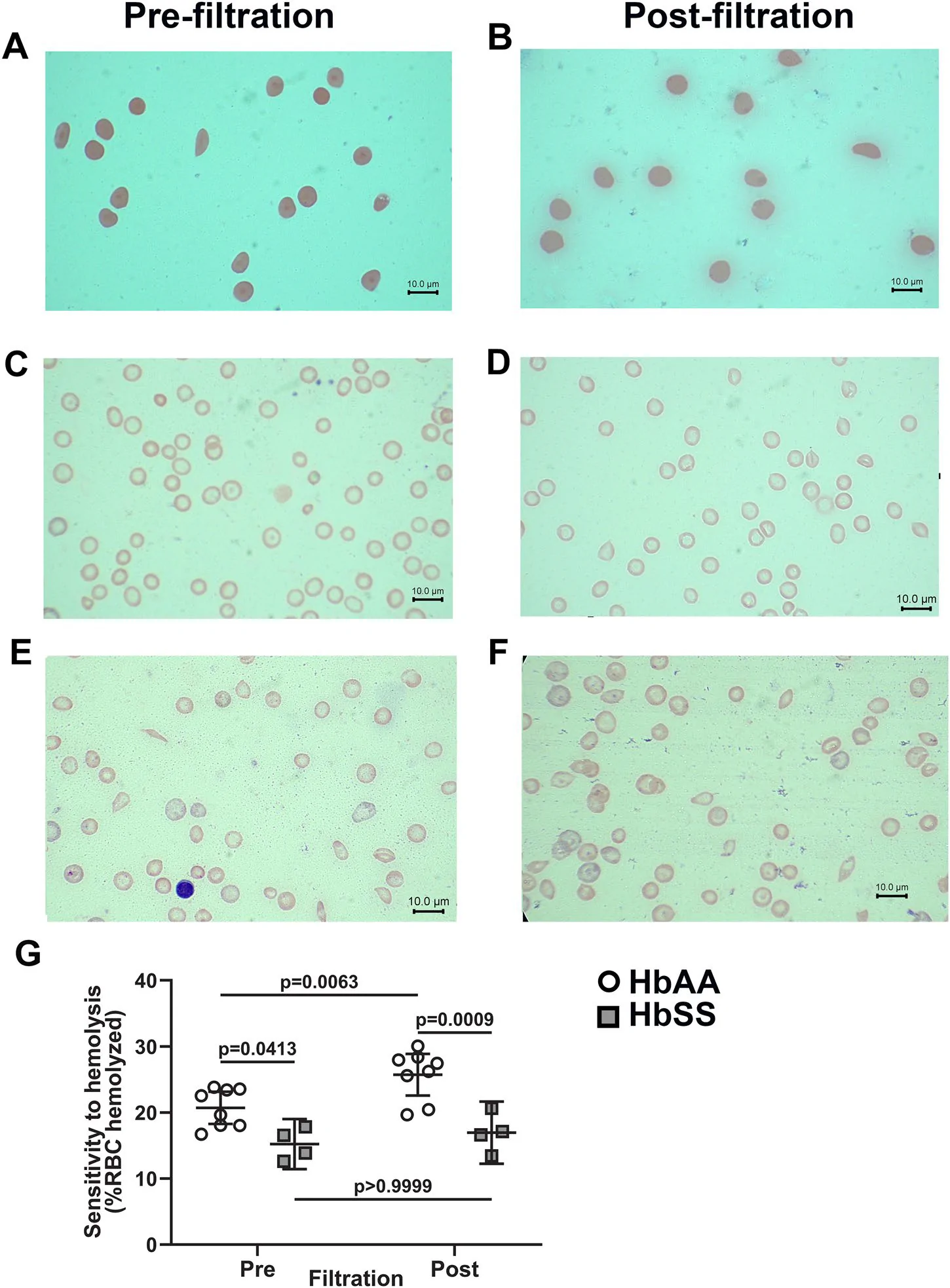
RBC morphology remains intact after MCE filtration. Membrane filtration preserves overall shape and appearance of red blood cells (RBC). Blood smears were obtained before and after sample filtration, cells were staining with Wrigth-Giemsa and visualized with 100x oil-immersed microscopy. Left and right columns are representative images of samples pre- and post-filtration respectively. Representative smears of human (A and B), HbAA (C and D) and HbSS (E and F) samples. Pre- and post-filtration images are from the same respective sample. (G) We also examined RBC osmotic fragility as a surrogate measure of sensitivity to hemolysis of RBCs exposed to a hypotonic solution. With membrane filtration, there was an increase in sensitivity to hemolysis in HbAA, but not HbSS samples (p > 0.9999).

We also evaluated RBC recovery (Figure 4), morphology (Figure 5), sensitivity to hemolysis (Figure 5), and sample RBC composition (Figure 6) before and after filtration. With MCE filtration, 74% (71.9–76.2%, 95% CI) of RBCs from HbAA samples and 61.8% (56.1 – 67.4%, 95% CI) of RBCs from HbSS samples were recovered (Figure 4B) and these RBCs were morphologically similar to those pre filtration (Figure 5A–F). We also examined RBC osmotic fragility as a surrogate measure of sensitivity to hemolysis of RBCs exposed to a hypotonic solution. With membrane filtration, there was an increase in sensitivity to hemolysis (p = 0.0063) in HbAA, but not HbSS samples (p > 0.9999), Figure 5G.

We next examined the effect of membrane filtration on the relative proportions of immature (predominantly reticulocytes) and mature RBCs in both HbAA, which do not have reticulocytosis, and HbSS mice. RBC populations were analyzed by flow cytometry, with total RBCs defined as Ter119^+^ cells, immature RBCs as Ter119^+^ and CD71^High^, and mature RBCs as Ter119^+^ and CD71^Low^ (see material and methods). Before filtration, in HbAA blood, immature RBCs accounted for 7.6% (5.3–9.7%) [mean (95% CI)] compared with 30% (18.4–41.5%) in HbSS, Figure 6A. Membrane filtration of HbAA samples yielded a trend for reduction in immature RBCs that did not reach significance. Conversely, filtration of HbSS blood yielded significant reduction in immature RBCs to 23% (12–35.2%), p < 0.0001, Figure 6A, indicating that membrane filtration modestly but significantly reduced the immature RBCs fraction in HbSS blood. Consistent with these findings, membrane filtration resulted in corresponding modest but significant increases in the proportion of mature RBCs in both HbAA and HbSS mice (Figure 6B), reflecting an enrichment of mature RBC population. Finally, membrane filtration significantly reduced the reticulocyte-to-mature RBC ratio in HbSS mice (p < 0.0001), whereas no significant change was observed in HbAA mice (p = 0.6655; Figure 6C).

Overall, membrane filtration resulted in a modest yet statistically significant reduction in reticulocytes in blood samples exhibiting reticulocytosis, an effect not reported with cellulose-based filtration [5]. Because reticulocytes are generally larger and less deformable than mature RBCs [13], their partial depletion is likely due to mechanical retention within the membrane, although nonspecific interactions between reticulocyte surface molecules and the MCE membrane cannot be excluded.

### 3.5. Filtered, purified RBCs are functionally intact

**Figure 7.**
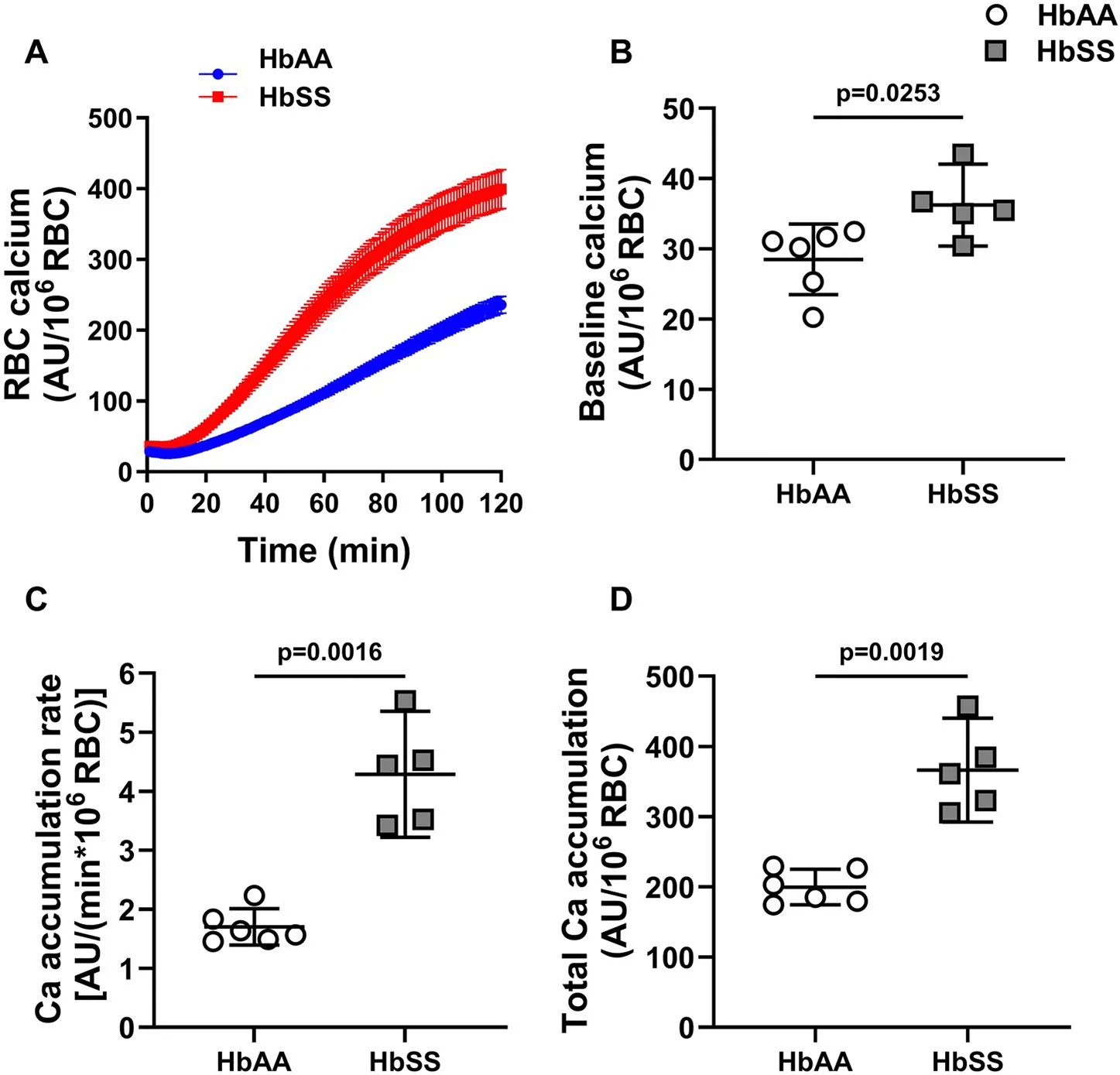
Purified RBCs are functional. (A) Data are shown as least square means ± SEM (N = 6 HbAA and 5 HbSS). As previously reported [11], humanized (HbAA) or sickle (HbSS) red blood cells spontaneously accumulate intracellular calcium. (B). HbSS cells have higher baseline calcium (p = 0.02); (C) higher calcium accumulation rate (p < 0.0001) and (D) higher calcium levels after 100 min (endpoint) (p = 0.002). In B, C, and D, data are shown as individual mouse measurements with superimposed least square means and 95% confidence interval. HbAA, N = 6 and HbSS, N = 5.

Once we established the purity of filtered samples, we investigated the integrity of RBC biochemical functions. We examined RBC calcium flux (Figure 7) to compare the findings of RBC purified with membrane filtration with those using cellulose filtration—the established gold standard for RBC purification. Our previous work showed that HbSS RBCs purified via cellulose filtration exhibit elevated baseline calcium levels, faster calcium accumulation, and higher peak intracellular calcium concentrations compared to HbAA RBCs [10,11]. In agreement with observations in RBCs purified with cellulose filtration, membrane-filtered HbSS RBCs had higher baseline calcium (p = 0.0253, Figure 7B), calcium accumulation rate (p = 0.0016, Figure 5C) and total calcium accumulation (p = 0.0019, Figure 7D) compared with HbAA RBCs.

Together these findings indicate that membrane-filtered RBCs remained functional exhibiting calcium accumulation comparable to that observed in cellulose-filtered RBCs [11], underscoring preserved physiological integrity of the purified RBC population.

## 4. Conclusion

Leukocyte reduction by filtration is well established in transfusion medicine and laboratory practice, with commercially available systems primarily designed for large-volume blood processing and leukocyte capture. Here, we describe a simple, low-cost approach tailored for small-volume RBC purification suitable for experimental applications, including human samples and murine models. Using MCE membranes, we demonstrate substantial depletion of leukocytes (~99%) and platelets (~90%), while preserving RBC populations. A key feature of this approach is the ability to adjust membrane pore size to specific sample types, thus providing flexibility not readily achievable with fixed cellulose-based filtration systems. We observed that optimal filtration conditions vary depending on species and RBC phenotype, with different pore sizes required for human versus murine samples, as well as for healthy and sickle cell blood. While this flexibility may be advantageous in studies involving heterogeneous RBC populations, filtration efficiency is influenced not only by cell size but also by cell deformability and structural heterogeneity. These features may pose limitations as less deformable or structurally distinct RBC subpopulations may be preferentially retained during filtration, thereby altering the representation of specific RBC subpopulations. Nevertheless, overall, our findings support the utility of MCE membrane filtration as a practical, low-cost and accessible tool for RBC enrichment in small-volume experimental settings.

## 5. Future perspectives

The membrane-based RBC purification approach described here provides a versatile platform that can be further optimized and expanded to meet emerging needs in RBC research and translational applications. The availability of commercially manufactured membranes with defined and reproducible specifications opens opportunities for cross-laboratory standardization, which can improve reproducibility across RBC research studies and support comparative analyses across species especially in settings using low-volume samples.

## Data Availability

Research data is available upon reasonable request to the authors.

## HIGHLIGHTS

- A simple membrane-based filtration method enables efficient purification of red blood cells without specialized equipment.
- The approach achieves robust leukoreduction and platelet removal in both human and murine blood samples.
- Adjustable membrane pore sizes allow customization for different species and RBC deformability states.
- The method provides flexibility not readily achievable with cellulose-based filtration systems, which have fixed pore size and reduced efficiency when processing diseased RBCs.
- This practical and low-cost technique supports reliable downstream biochemical and functional RBC analyses in laboratory research.

## References

[1] King KE, Shirey RS, Thoman SK, Universal leukoreduction decreases the incidence of febrile nonhemolytic transfusion reactions to RBCs. Transfusion. 2004;44(1):25–29. doi: 10.1046/j.0041-1132.2004.00609.x 14692963

[2] Cervia JS, Wenz B, Ortolano GA. Leukocyte reduction’s role in the attenuation of infection risks among transfusion recipients. Clin Infect Dis. 2007;45(8):1008–1013. doi: 10.1086/521896 17879916

[3] Minetti G, Egée S, Mörsdorf D, Red cell investigations: art and artefacts. Blood Rev. 2013;27(2):91–101. doi: 10.1016/j.blre.2013.02.002 23425684

[4] Ciana A, Achilli C, Balduini C, On the association of lipid rafts to the spectrin skeleton in human erythrocytes. Biochim Biophys Acta. 2011;1808(1):183–190. doi: 10.1016/j.bbamem.2010.08.019 20807499

[5] Beutler E, West C, Blume KG. The removal of leukocytes and platelets from whole blood. J Lab Clin Med. 1976;88(2):328–333. 956688

[6] Beutler E, Gelbart T. The mechanism of removal of leukocytes by cellulose columns. Blood Cells. 1986;12(1):57–64. 3790738

[7] Gautier EF, Leduc M, Cochet S, Absolute proteome quantification of highly purified populations of circulating reticulocytes and mature erythrocytes. Blood Adv. 2018;2(20):2646–2657. doi: 10.1182/bloodadvances.2018023515 30327373 PMC6199659

[8] Wang L, Almeida LEF, de Souza Batista CM, Cognitive and behavior deficits in sickle cell mice are associated with profound neuropathologic changes in hippocampus and cerebellum. Neurobiol Dis. 2016;85:60–72. doi: 10.1016/j.nbd.2015.10.004 26462816 PMC4688201

[9] Kamimura S, Smith M, Vogel S, Mouse models of sickle cell disease: Imperfect and yet very informative. Blood Cells Mol Dis. 2024;104:102776. doi: 10.1016/j.bcmd.2023.102776 37391346 PMC10725515

[10] Almeida LEF, Smith ML, Kamimura S, Metabolic stimulation improves bioenergetics and haematologic indices of circulating erythrocytes from sickle cell mice. J Physiol. 2025; doi: 10.1113/JP287673 39775917

[11] Almeida LEF, Smith ML, Kamimura S, Calcium flux alterations in erythrocytes from sickle cell mice: The relevance of mean corpuscular volume. Blood Cells Mol Dis. 2024;104:102800. doi: 10.1016/j.bcmd.2023.102800 37951090 PMC10842784

[12] Achilli C, Ciana A, Balduini C, Application of gelatin zymography for evaluating low levels of contaminating neutrophils in red blood cell samples. Anal Biochem. 2011;409(2):296–297. doi: 10.1016/j.ab.2010.10.019 20971053

[13] Chasis JA, Prenant M, Leung A, Membrane assembly and remodeling during reticulocyte maturation. Blood. 1989;74(3):1112–1120. doi: 10.1182/blood.V74.3.1112.1112 2752157

